# Towards circular healthcare: A qualitative exploration of approaches and implementation factors in Dutch hospitals

**DOI:** 10.1016/j.joclim.2026.100727

**Published:** 2026-10-01

**Authors:** Maaike L. Soors d’Ancona, Óscar Brito Fernandes, Ans Kolk, Lisanne H.J.A. Kouwenberg, Nicolaas H. Sperna Weiland, Dionne S. Kringos

**Affiliations:** a Amsterdam UMC location University of Amsterdam, Public and Occupational Health, Meibergdreef 9, Amsterdam, The Netherlands; b Amsterdam Public Health research institute, Quality of Care, Amsterdam, The Netherlands; c Amsterdam UMC, Centre for Sustainable Healthcare, Amsterdam, The Netherlands; d Amsterdam Business School, University of Amsterdam, The Netherlands; e Amsterdam UMC location University of Amsterdam, Department of Anesthesiology, Meibergdreef 9, Amsterdam, The Netherlands

**Keywords:** Healthcare, Climate change, Environmental impact, Environmental sustainability, Circularity, Implementation science

## Abstract

**Background:**

Healthcare is a major contributor to environmental pollution, mainly due to high resource use and a linear supply chain. In the Netherlands, healthcare consumes 13% of all abiotic raw materials and produces 4.2% of national waste. Although optimizing resource use and minimizing waste through circularity seems the way forward, it remains poorly understood. This study (1) explores how hospitals in the wider Amsterdam region approach circularity, and (2) identifies factors influencing implementation.

**Methods:**

Semi-structured interviews, informed by an implementation framework, were conducted with hospital staff in roles relevant to circularity implementation. Interviews continued until data saturation. Verbatim transcripts were coded and thematic analysis was applied.

**Results:**

Nineteen staff from twelve hospitals were interviewed. Institutionalization of circularity varied widely, from structured approaches with strategies, coordination, monitoring, and budgets to more fragmented, project-based efforts driven by individual champions. Key factors influencing implementation included bottom-up motivation, top-down support, infection prevention, internal and external collaboration, supply-chain dependency, policies, and regulations.

**Conclusion:**

Hospitals in the wider Amsterdam region are motivated to contribute to the circular transition, with staff organizing green teams and other bottom-up initiatives with varying levels of organizational support. Yet bottom-up momentum alone is insufficient. Because environmental benefits of circularity accumulate largely at the societal level while hospitals bear operational, financial, and infection-prevention risks, progress requires stronger top-down engagement and system-level changes. Additionally, regulations and policies need to address the absence of affordable circular alternatives, dependence on incumbent linear-economy suppliers, and showcase co-benefits of circularity to hospital management, regulatory bodies, and health insurers.

## Graphical abstract

**Figure:**
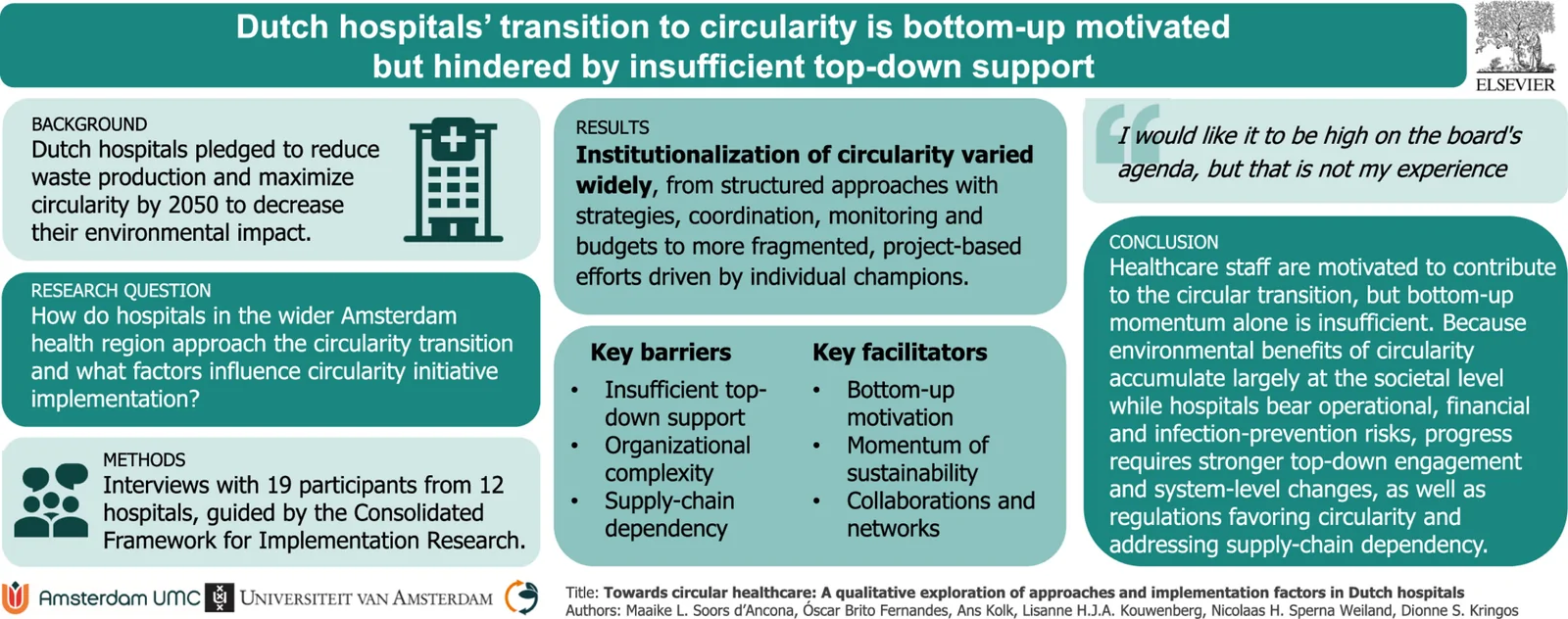

## 1 Introduction

Climate change is detrimental to human health, yet healthcare also contributes to the problem. Healthcare systems contribute 4.4–5% of global greenhouse gas emissions, a share rising to nearly 10% in high-income countries [1,2]. In these countries, the vast majority of emissions stem from scope 3: the supply chain [3]. In the Netherlands, the healthcare sector contributes approximately 7.3% of the national carbon footprint. This impact is largely driven by a linear healthcare economy−also known as a "take, make, dispose" system wherein resources are extracted to make products that are used and eventually disposed of as waste−generating 4.2% of national waste and using 13% of all abiotic raw materials. In contrast, a circular economy is a system that focuses on reducing, reusing, recycling, and recovering materials [4], thereby reducing waste, enhancing supply chain resilience, and decreasing environmental harm [5]. This highlights the urgent need for circular strategies, such as reducing unnecessary material use, and transitioning from disposable to reusable equipment, particularly in hospital settings [6]. By 2022, all Dutch hospitals had signed the 'Green Deal 3.0', and within its circularity pillar they have committed to halving primary raw material use by 2030 and achieving maximum circular healthcare by 2050 [7]. This pledge can be seen as a first step towards a circular economy in the Dutch healthcare system.

Growing evidence shows that circular healthcare practices, including switching to reusable medical products and reducing material use, can significantly reduce environmental impacts [[8], [9], [10], [11], [12], [13], [14]]. Yet these studies primarily assess environmental performance or compare specific products. Much less is known about how hospitals operationalize circularity in complex organizational settings, where decision-making processes, professional norms, supply chain constraints, and regulatory requirements interact.

Important knowledge gaps remain regarding how factors inside hospitals, such as the roles and actions of key actors and organizational structures, and external factors, such as market dynamics and regulatory requirements, influence the adoption, scaling, and long-term sustainability of initiatives. Moreover, implementation science has rarely been applied in this context, leaving important questions about how hospitals can progress from pilots to the institutionalization of circular practices across departments, in organization-wide processes, and towards supply chains [15].

This study aims to (1) provide an overview of ways in which Dutch hospitals are approaching circular healthcare, and (2) identify factors that influence the implementation of circular initiatives. The insights inform strategies for embedding circular practices in hospitals and contribute to more sustainable and circular hospitals and healthcare systems.

## 2 Methods

### 2.1 Study design and data collection

From February to July 2025, semi-structured interviews were conducted with staff from hospitals in the wider Amsterdam region (The Netherlands) in roles relevant to circularity implementation. The interview guide was informed by the updated Consolidated Framework for Implementation Research (CFIR) (Appendix A), which enabled the collection of nuanced insights. Specifically, the CFIR process domain, complemented by additional open-ended questions, addressed how hospitals approach circular healthcare, while all CFIR domains informed the identification of factors influencing implementation processes (Appendix B). Documents including policy and strategy documents on environmental sustainability were requested from participants prior to the interview via email. Desk research of these documents and hospital websites was used to inform the interviews. Data collection continued until no new insights were identified from the interviews, indicating data saturation. We adhered to the consolidated criteria for reporting qualitative studies (COREQ) (Appendix C, Table C.1).

### 2.2 Ethical considerations

Ethical review was conducted by the non-WMO Committee of the Medical Ethics Review Committee of Amsterdam University Medical Center (IRB00013752, document number: 2025.0113), which concluded this study falls outside the scope of the Medical Research Involving Human Subjects Act (WMO; Wet Medisch-wetenschappelijk Onderzoek met mensen). Written or verbally-recorded informed consent was obtained prior to the interviews.

### 2.3 Participant selection

Using a purposive sampling approach, study participants were recruited from hospitals in an existing regional healthcare network, which includes but is not limited to the ROAZ (Regionaal Overleg Acute Zorg) acute care network of region Noord-Holland/Flevoland, here referred to as the wider Amsterdam region [16]. Eligible participants (1) held roles relevant to circularity implementation (e.g., sustainability coordinators/advisors, medical specialists, infection prevention specialists, support services staff) and (2) had held their role for at least six months. The research team members leveraged their professional networks to recruit relevant participants. Recruitment was subsequently expanded via snowball sampling, whereby participants suggested other suitable participants. Potential participants received written information beforehand. Interviews of approximately one hour were scheduled in person or online, depending on participants’ availability.

### 2.4 Interview conduct

Interviews were audio recorded with or without video, depending on participants’ preferences, then automatically transcribed verbatim using Microsoft Teams [17], and subsequently checked manually for accuracy. During the interviews, participants were asked to share their experiences regarding the hospital’s circular transition approaches and implementation of circular initiatives, following a semi-structure interview guide. Key insights and potential quotations were sent to participants for corrections and validation. Quotations were referenced by participant number and role to ensure confidentiality.

### 2.5 Data analysis

MAXQDA software was used for coding and data analysis [18]. Transcripts of the first two interviews were independently coded by two researchers (MS, LK) to enhance the depth and nuance of data interpretation. The remaining transcripts were coded by MS. Transcripts were deductively coded to identify barriers and facilitators of circularity implementation, using the updated CFIR [19]. Open coding was employed to capture hospitals’ approaches to circular healthcare. The codebook was updated iteratively throughout the analysis. Interactions and dynamics between the identified barriers and facilitators were explored. This thematic analysis entailed examining patterns, connections, and influences across CFIR domains, to generate themes that extended beyond the original CFIR structure.

## 3 Results

### 3.1 Overview of participating hospitals and participants

Nineteen staff members from twelve hospitals were included (Table 1). The average interview duration was 47 minutes. Fifteen were held online, and four in person.

**Table 1:** Hospital and participant characteristics.

**Characteristics**		**N (%)**
**Hospitals**		Total = 12
Type	General	10 (83%)
	Specialized	1 (8%)
	Academic	1 (8%)
Hospital locations*	1	7 (58%)
	≥2	5 (42%)
**Participants**		Total = 19
Role	Medical specialist	7 (37%)
	Sustainability coordinator	5 (26%)
	Sustainability advisor	3 (16%)
	Support services staff	2 (11%)
	Infection prevention specialist	2 (11%)

⁎ Number of physical hospital sites belonging to the participating organization.

### 3.2 Hospitals’ approaches to circular healthcare

Participants reported several approaches in their respective hospitals towards operationalizing circular healthcare (Table 2). Green teams and sustainability committees, coordinators and advisors were frequently present, dedicating time to implement initiatives and increase sustainability awareness and knowledge. Circularity was differently integrated into strategies and policies, ranging from general intentions to concrete commitments. There was great variation between hospitals regarding dedicated sustainability budgets, with most hospitals lacking such a budget. Across hospitals, circular efforts were highly fragmented and largely project-based, with most initiatives emerging from motivated individuals or green teams rather than from a structurally-embedded organizational program. Only a minority of hospitals monitored progress using quantitative data systematically. Lastly, all hospitals entered into external collaborations and/or networks, such as collaborations with other (regional) hospitals.

**Table 2:** Hospitals’ approaches to circular healthcare.

*A. Key roles and teams involved in circularity implementation*				
Roles/teams	Description	Uptake and variation	Main roles and actions	Illustrative quote
**Green teams**	Teams of medical specialists and other healthcare staff dedicating time to sustainability practices, usually organized at department level.	Present in all hospitals, ranging from one to dozens of teams.	• Gather ideas for initiatives • Implement (primarily small-scale) initiatives	“The five largest [department green teams] always know where to find us, but they are also working on the largest projects. The smaller green teams often look for low-hanging fruit, which mainly involves purchasing policy and minor changes in work processes, and they always try to test these with us.” – Participant 17, infection prevention specialist
**Sustainability coordinators and advisors**	Staff responsible for the coordination and organization of the hospital’s sustainability efforts.	Present in 9 hospitals.	• Gather ideas of initiatives • Support green teams • Oversee ongoing projects • Create awareness and provide education on circular workflows • Initiate and supervise pilots • Write business cases for initiatives	“I do a lot of training, informing the green teams about why it's important, what the challenge is, how it affects healthcare, how we can contribute to a solution, and how that translates into the hospital's goals. And additionally I help with prioritizing” – Participant 10, sustainability coordinator
**Sustainability committees**	Multidisciplinary committee, often including sustainability coordinator or advisor and support services staff, usually organized at a hospital level.	Present in 10 hospitals.	• Address larger scale initiatives and hospital-wide challenges • Contribute to policy and strategy development • (Often) have decision-making power at hospital level	“The idea was that we, as the sustainability committee, wanted to raise awareness in the hospital, but we also wanted to support the green teams, so we encouraged every department to set up a green team. We were successful in this, and ultimately incorporated a sustainability plan into the strategy” – Participant 6, medical specialist

*B. Organizational approaches*				
Approach	Description	Uptake and variation	Main roles and actions	Illustrative quote
**Integrating circularity in strategies and policies**	Integrating circularity in the hospital’s overall policy plans, strategies, and procurement policies.	Present in all hospitals, though with inter-hospital differences, ranging from non-committal to concrete commitments.	Integrate sustainability in: • Hospital strategy • Overall policy plan • Procurement policy	“I also think that our organization is quite progressive in this regard, in that it has made it a pillar of its policy. Most hospitals have four standard pillars, right? Patient care, employee satisfaction, et cetera, but we also have the pillar of sustainability, as in, guys, we just want to reach our goals” – Participant 17, infection prevention specialist
**Creating a dedicated sustainability budget**	Creating a budget dedicated to sustainability, either structurally, temporary, or through shared-savings.	No budget (n = 8), temporary budget (n = 2), structural and shared-savings budget (n = 1), sporadic shared-savings budget (n = 1).	• Invest in reusables or less environmentally harmful equipment • Allocate time for green team members or related staff • Hire sustainability coordinators	“In addition [to a budget], [we] introduced shared-savings, so if we save money, […] we've stopped using scrub sponges, or, well, we stopped doing certain things or we reduce them, then half of those savings go into the sustainability budget, so that we can finance other things that cost more money” – Participant 4, sustainability advisor
**Creating awareness and knowledge campaigns**	Dedicating time to raise sustainability awareness and knowledge among healthcare staff, usually by sustainability coordinators and advisors.	Present in all hospitals.	• Provide education • Write newsletters • Create competitions • Share successes • Write annual reports • Visualize waste • Show potential win-win benefits	“Circularity is, of course, a difficult subject, but it has a lot to do with support and awareness, and ultimately it is support and awareness that I need to achieve among my fellow healthcare workers. If I succeed in doing that, then the chances of success are many times greater” – Participant 2, sustainability coordinator
**Monitoring progress with quantitative data**	Monitoring circular progress via quantitative data on reduction of (unsorted) waste, use of raw materials and/or disposables.	Present in 4 hospitals, of which 1 reported using a dedicated dashboard.	• Conduct waste stream analyses • Collect quantitative data on (unsorted) waste, raw material and/or disposable use • Dashboard use • Compare results to 'Green Deal 3.0' circular goals	“In the dashboard, you can see [our progress] in relation to those goals. And the specific goals we have set there are the same as those in the Green Deal” – Participant 8, sustainability coordinator
**Entering into external collaborations and networks**	Entering into collaborations of healthcare staff with external parties to share knowledge about initiatives and experiences with implementation strategies, to divide circular projects between hospitals and aiming to create market power.	Collaborations of healthcare staff with external commissions, working groups, associations and/or networks (n = 10), other (regional) hospitals (n = 11), and supply-chain actors (n = 6).*	Collaborate with: • External commissions, working groups, associations, and networks • Other hospitals • Supply chain actors	“[We are] in a network where specific areas of focus are divided among the hospitals […] so we can learn from each other. But we also need scale for some things, such as the washable surgical gowns. The more hospitals that do this, the cheaper it becomes” – Participant 4, sustainability advisor

⁎ The total exceeds the number of hospitals (n = 12) because individual hospitals reported multiple types of collaborations.

### 3.3 Factors influencing implementation

Eight themes were generated to map both barriers and facilitators in the implementation of circular initiatives: (1) resource availability, (2) implementation process, (3) circular initiative characteristics, (4) staff and leadership motivation, engagement and attitudes, (5) tension between infection prevention and circularity, (6) organizational complexity, (7) regulations, certification and policy, and (8) external stakeholders and suppliers (Table 3).

**Table 3:** Key implementation barriers and facilitators organized into 8 overarching themes.

**Barriers**	**Facilitators**
*Theme 1: Resource availability*	

• Implementation costs • Lack of budgets • Shared-savings issues • Insufficient dedicated sustainability hours • Absence of sustainability coordinator • Workforce capacity • Insufficient expertise • Shortage of storage space	• Dedicated budgets • Dedicated hours to sustainability • Presence of sustainability coordinator • Internal and external knowledge sharing • Potential advantage for attracting personnel • Strong business cases

*Theme 2: Implementation process*	

• Slow process hindering enthusiasm	• Structured implementation plan • Timely involvement of relevant internal stakeholders • Repeated exposure to initiative

*Theme 3: Circular initiative characteristics*	

• Missing data on sustainability impact • Less user-friendly initiative • Affecting personal expression/identity • Requiring more time and effort • Low product volume	• Data proving sustainability impact • Matched or improved workflow and personal preferences • Same price or saving costs

*Theme 4: Staff and leadership motivation, engagement and attitudes*	

• Staff’s resistance to change • Circularity perceived as added work burden • Skepticism about impact • Sustainability not part of hospital culture • Insufficient top-down prioritization, motivation, and support	• Momentum around sustainability • Wanting to keep up with other hospitals • Pioneering initiatives • Motivated green team members and individual champions • Collegial and managerial motivation and support

*Theme 5: Tension between infection prevention and circularity*	

• Infection prevention protocols and guidelines • Initiatives perceived as complex or high-risk • Late involvement in implementation process • Dependence on national guidelines • Lack of or unfavorable guidelines	• “Green” infection prevention specialists • Data proving safety of initiatives • Updated guidelines in favor of circularity

*Theme 6: Organizational complexity*	

• Large organizational sizes • Short-term budget focus • Managing differences across locations • Many stakeholders and organizational layers • Imposing measures perceived as incompatible with the hospital environment	• Small organizations/locations with short lines of communication

*Theme 7: Regulations, certification and policy*	

• (Inter)national regulations insufficiently considering environmental impact • Lack of acting on hospital policy or strategy	• Potential regulatory changes • Certification incentivizing sustainable practices • Having clear top-down goals or policies

*Theme 8: External stakeholders and suppliers*	

• Supply chain dependency • Suppliers not sharing product data • Obstructive or ignorant suppliers • Greenwashing • Scale needed for affordability • Outsourcing capacity and pricing issues • Hospitals having many existing procurement contracts	• Willingness of (new) suppliers to address sustainability • Leveraging hospital collaborations for market power • Higher product volumes raising suppliers’ interests

Note: For a complete overview, Table D.1 (see Appendix D) contains all barriers and facilitators, categorized according to CFIR domains and organized into the eight themes.

#### 3.3.1 Theme 1: Resource availability

Budget was reported as an important factor. Circular initiatives often require investments, especially when switching from disposable to reusable products. Hospitals without structural budgets struggled to move beyond small projects, whereas those with dedicated funds reported clear advantages. Some hospitals attempted shared-savings systems (e.g., reallocating money saved by reducing disposable use), but faced difficulties in tracking costs and departmental reluctance to transfer savings. Many hospitals use business cases for decision-making processes, and strong business cases were advantageous, especially when costs were involved.

Time was also a major constraint, especially in hospitals without sustainability coordinators. Several participants mentioned a lack of dedicated hours, slowing implementation. Dedicated hours for green team members, sustainability coordinators, and advisors were considered important, but allocation was not structurally organized across departments and hospitals. Managers were often reluctant to involve staff due to ongoing workforce shortages and concerns about higher workload, dissatisfaction, and staff leaving. Capacity constraints in infection prevention, sterilization, and purchasing also hindered implementation. However, several hospitals tried to leverage their sustainability efforts to improve competitiveness and attract staff amid shortages.

Space was another commonly mentioned barrier. Circularity is perceived as often requiring additional storage rooms, for example for waste separation bins. Most hospitals have difficulty organizing extra space due to the physical limits of their facilities. Some tried to address this with assortment coordinators to optimize product storage, by creating additional space during renovations, or by using waste compactors.

Participants also noted insufficient expertise in green teams to implement large-scale, bottom-up projects, as well as a lack of vision on what constitutes “maximum circular healthcare”. A similar challenge was insufficient expertise of circularity among general healthcare staff, and the assumption that sustainability is inherently expensive, both hindering progress. Conversely, collaborations between hospitals were recognized as key facilitators, to share best practices and information on initiatives, and to prevent duplicative efforts. Additionally, online platforms, professional associations, and national committees were perceived valuable for knowledge exchange and motivation. While green team meetings and internal education and awareness campaigns helped bridge knowledge gaps, efforts tended to reach staff already interested in sustainability. An example of effective awareness-building was the hospital-wide reduction of disposable cellulose mats, which showed both environmental and financial benefits:

*“Look, regarding those cellulose mats, we are now undertaking a huge project, throughout the entire hospital, which took us two months to reduce the use of [disposable] cellulose mats. And we've actually achieved a reduction of, well, ∼30% hospital-wide. And even ∼40% if you add up several years, […], we really use so many [disposable] cellulose mats, so that awareness has definitely had an impact, and by reducing that, you naturally also have lower costs” – Participant 11, sustainability coordinator*

#### 3.3.2 Theme 2: Implementation process

Participants noted the importance of a structured implementation plan, including baseline measurements, concrete goals, and small-scale pilot implementation of circularity initiatives before wider rollout, while avoiding unnecessarily slow processes that could reduce initial enthusiasm. This also applied to timely involvement of relevant internal stakeholders, including direct users, procurement departments, and facility services. Ensuring continued communication concerning upcoming initiatives additionally facilitated acceptance.

Participants reported a mix of incremental and radical implementations, showing different preferences. For example, while some chose to still provide the old disposable product alongside the reusable one for back-up purposes, others radically moved toward the new product as this reportedly creates quicker impacts with more straightforward implementation.

#### 3.3.3 Theme 3: Circular initiative characteristics

While data proving sustainability impact facilitated implementation, this was not always present, causing difficulties in providing evidence.

*“We simply had a life cycle analysis, which enabled us to conclude based on evidence that, when considering the entire process, including washing and detergent, which was naturally everyone's argument, […] then you could just say based on that data like no, yeah, when considering the entire process, then it is indeed sustainable, which was very helpful to us at the time” – Participant 6, medical specialist*

Initiatives perceived as less user-friendly, such as certain reusable medical equipment and washable clothing, faced implementation challenges. In contrast, initiatives that aligned with staff preferences, like washable heated blankets, were more easily adopted. Resistance arose when initiatives affected personal expression/identity via changes in work clothing, particularly when replacing disposable surgical headcovers with reusable ones.

Initiatives that disrupted existing workflows also negatively affected implementation. Transitions from disposables to reusables and waste separation often require more work and logistical adjustments. In contrast, initiatives that align with or improve workflows, such as using less harmful disposables, were more successful. Some reusable products involved barriers due to low volume which limited feasibility; collaboration between hospitals was noted to help scalability. When initiatives reduced costs, some participants perceived the implementation as less challenging.

#### 3.3.4 Theme 4: Staff and leadership motivation, engagement and attitudes

Participants observed widespread growing momentum around sustainability and a subsequent need for circular practices, triggering increased interest of hospital staff, health insurers, and other actors involved in the supply chain. Furthermore, many hospitals seem motivated to keep up with others, publicly showcase their progress, and pioneer initiatives. Motivated healthcare staff and support from peers, department heads, and support services were important for implementation success, while demotivation often arose from slow processes and negative colleague attitudes. Broad staff engagement was essential, though resistance to change due to perceived higher workload, skepticism, and generational gaps created challenges. Effective engagement strategies included friendly competition and positive framing. Despite momentum, sustainability was not yet perceived as embedded in hospital culture, and top-down leadership support and priority to the topic was often flagged as lacking. Participants noted a lack of centralized, top-down guidance, and board-level engagement as major challenges, since board-level support was crucial for organization-wide decisions on circularity, including budget allocation and policy changes. Efforts often relied on individual, bottom-up motivation, which tended to fade without structural top-down support.

*“Now, from bottom-up arises a lot, quite a lot of motivation in the initiatives, but if they are not supported top-down, structurally, then that will fade away. So even though we are included in the strategy, we are not really part of the organization, […] so you have to find your own way instead of it being there for you, or something like that” – Participant 6, medical specialist*

#### 3.3.5 Theme 5: Tension between infection prevention and circularity

The tension between circularity and infection prevention protocols and guidelines was commonly mentioned as a barrier, especially in perceived high-risk areas such as the operating room. Having “green” infection prevention specialists actively engaged was considered helpful, and those with this expertise emphasized the importance of being involved early to avoid unnecessary work and setbacks:

*“[…] projects were already starting up in the hospital, and a lot of work had already been done, and only then were we asked, and then we had to say, ‘No, that's not allowed,’ and that was such a waste of work” – Participant 16, infection prevention specialist*

The presence of data proving safety or quality standards of circular products facilitated implementation, but this was often lacking. Therefore, reducing unnecessary material use was sometimes prioritized over switching to reusables. Infection prevention decisions were noted to highly depend on national guidelines, which participants mentioned lacked sufficient consideration of circularity. Updated national guidelines in favor of circularity were therefore considered essential. Participants noted that general hospitals in particular benefit from such updates, as they tend to rely more heavily on national guidelines. While also adhering to them, academic hospitals often do their own targeted research and tailor implementation accordingly.

#### 3.3.6 Theme 6: Organizational complexity

The complexity of a hospital organization was mentioned as a major barrier. Challenges included a short-term budget focus, managing differences across locations, frequent staff turnover, dealing with many stakeholders and organizational layers, and difficulties identifying initiative leaders. Smaller hospitals reported smoother implementation due to shorter communication lines, while larger hospitals frequently faced obstacles due to their size.

*“I find the whole organization very difficult when it comes to getting things done. I find it very slow-moving within the healthcare sector. And I hear the same thing from other hospitals, but everyone has something to say about everything and you can't take everyone into account, and if you do, well, then it will take months before a decision is made” – Participant 11, sustainability coordinator*

Some hospitals only implement initiatives hospital-wide to ensure uniform procedures, requiring initiatives’ approval from many departments in case of decentralized decision processes. Additionally, imposing sustainability was seen as incompatible with the hospital environment, often leaving sustainability coordinators with insufficient decision-making power. Still, progress was visible: one department head noted that sustainability has become part of routine work and no longer felt like an extra task.

#### 3.3.7 Theme 7: Regulations, certification and policy

Some participants criticized (inter)national medical regulations’ insufficient consideration of environmental impact. For example, the European Medical Device Regulation was noted to contain a number of barriers that complicates the circular transition. There was a desire for sustainability regulation for suppliers, as participants saw suppliers but also hospitals, banks, and healthcare insurers being positively triggered by then upcoming regulations. To illustrate, the European Union's Corporate Sustainability Reporting Directive (CSRD, announced at the time but not fully approved yet) led one hospital to seek environmental certification, which incentivized institutionalization.

Participants noted the contribution of the 'Green Deal 3.0' to the integration of circularity in hospital policies and strategies, but insufficient follow-up and actionability were reported as major barriers. Several participants emphasized the importance of having clear sustainability goals from top management as part of long-term policy, to guide and justify changes, with boards seeing it as strategic priority instead of mere compliance.

*“The board of directors thinks it's important, but more as in whether we are complying with the laws and regulations” – Participant 7, sustainability coordinator*

#### 3.3.8 Theme 8: External stakeholders and suppliers

Supply chain dependency was mentioned as a major barrier, especially when switching to (usually more expensive) reusable products, frequently from different companies. While some existing suppliers willingly discussed their products’ environmental footprints, others were seen as rather reluctant to provide clear information, particularly with alternative (reusable) suppliers entering the market. Participants mentioned product quality marks as a possible solution, also to help combat greenwashing.

*“I think that manufacturers will resist because they have a financial interest in non-sustainable solutions, and we need to get rid of that. And I think that some employees and some manufacturers are really focused on sustainability, but the big manufacturers are just focused on making money, and they won't be so easy to get on board” – Participant 3, medical specialist*

Barriers furthermore included (existing) disposable suppliers decreasing prices after noticing the shift towards reusables, to try to “block” alternatives’ entrance. Since low product volumes prevent competitive pricing, hospital collaborations were sometimes leveraged to increase market power for circular alternatives and get better prices from suppliers. However, existing contracts and/or differences in contracts between hospitals complicated switching suppliers.

Hospitals also faced complexities working with waste processors, external laundries, and sterilization companies. Effective waste separation was hindered by refusal of waste processors to process limited waste volumes or contaminated streams, and by certain municipalities ending at-source waste separation which led to disincentivized staff and discontinuations. With outsourced laundry and sterilization, barriers included company capacity constraints and high prices due to market dominance of laundry suppliers.

## 4 Discussion

This study explored how Dutch hospitals are approaching circular healthcare and which factors influence its implementation. We found a range of approaches, including establishing new organizational structures, integrating circularity into strategies and policies, promoting awareness, and engaging in external collaborations. However, the degree of institutionalization varied markedly. Factors shaping implementation related primarily to bottom-up motivation, top-down support, collaborations, policies, regulations, and supply chain dependency. Our findings show that circularity represents a qualitatively different type of hospital change, sometimes disruptive, and not “business as usual”. It delivers predominantly external, societal value, rather than direct clinical or operational benefits, depends on multi-actor coordination across supply-chains, and often involves non-clinical workflows. Consequently, existing decision-making routines, incentives, and budgets are not yet aligned with circularity, and do not account for its (often longer-term and wider) benefits.

Following the signing of the 'Green Deal 3.0', hospitals have taken initial steps, including appointing sustainability coordinators, and forming green teams and sustainability committees. They reflect strong voluntary, bottom-up engagement with circularity, different from many other hospital change processes. Interestingly, such staff engagement appears relatively uncommon in many commercial enterprises (companies), where sustainability activities are more often guided through formal governance structures, regulations, certifications, and organizational strategies [20], with much less or no room for widespread bottom-up initiatives. Simultaneously, hospitals frequently lacked the top-down support required to turn these efforts into organization-wide change. Participants described limited budgets, insufficient protected time, non-committal strategies, and minimal quantitative progress monitoring.

Previous research highlights the importance of leadership, expertise, and funding for sustainable change in healthcare [[21], [22], [23]], and demonstrates that organization-wide strategies and performance monitoring are important for long-term success [24]. Our findings reinforce this; bottom-up motivation is an important facilitating factor, but without complementary top-down prioritization and investments, circularity remains fragmented and project-based rather than institutionalized. This dynamic mirrors early developments in the patient safety movement, where enthusiasm alone proved insufficient until measurement, transparency, accountability structures, and regulation were introduced [25].

Circularity appears to be at a similar inflection point, further inhibited by a lack of vision regarding what constitutes “maximum circular healthcare”. Hospitals therefore require stronger organizational mandates, reliable data, and systematic monitoring. Our findings also suggest that both the growing momentum of sustainability and potential upcoming regulations (e.g., the CSRD [26]) can stimulate interest not only among hospital staff but also among health insurers, banks, and supply chain actors. It should be noted that the CSRD, mentioned by one participant, was substantially reduced in scope in March 2026. Therefore, its importance at the time of the interviews may have overstated the current impact [27,28]. Instead, the upcoming European Union’s Circular Economy Act might stimulate the adoption of circular practices, also in healthcare [29].

Previous research also identified accreditation as necessary to advance sustainability, and the growing international interest in incorporating environmental sustainability into performance assessment frameworks may further support this shift [25,30]. An interesting development in this regard is that the hospital accreditation organization Joint Committee International (JCI) introduced a chapter on environmental sustainability in healthcare organizations in 2025, which is likely to lead to more attention for sustainability in the sector [31].

Workforce shortages emerged as a major contextual constraint. High workloads made managers reluctant to assign additional responsibilities, echoing research showing that sustainability is often deprioritized under clinical pressure [32,33]. Yet several circular strategies, such as optimizing surgical trays, can reduce unnecessary material use and simplify workflows [34]. Our interviews similarly revealed strong staff motivation to contribute to circularity, consistent with previous findings that nurses view sustainability as a professional duty [35]. Framing circularity as an opportunity rather than an added burden may therefore strengthen engagement and organizational alignment.

A notable barrier entailed the immaturity of circular supply-chain ecosystems, which are emergent and complex [36]. This contrasts with the existing disposable linear system, which is generally highly profitable for incumbent companies, with logistics optimized and easier compared to reusables (“deliver and throw away”). Participants mentioned reluctant suppliers, limited transparency, and price incentives favoring disposables. While some argue that verified environmental impact and safety data and clear reporting standards would help [25], the current “disposable lock-in” requires additional steps to build sustainable business models, and reduce hospitals’ supplier dependency [37]. Joint commitment to change, including pooled purchasing power, logistics, and knowledge are necessary to stimulate supplier responsiveness. Collaboration between hospitals can help to scale sustainable business models [38], with better prices and greater resilience to supply chain disturbances as co-benefits, as reusable products are said to be less sensitive to shocks [5].

Infection prevention played a dual role. Infection prevention requirements, often based on disposable workflows, were reported to conflict with circular practices [39]. Seen as a justification for maintaining disposable practices, participants noted that circular alternatives can meet safety standards when supported by evidence. Hospitals’ strong risk aversion means that even small uncertainties can halt innovation, underscoring the need for updated (inter)national guidelines that explicitly incorporate circular principles.

A recurrent topic was the asymmetry between benefits and risks. Environmental benefits of circularity accumulate largely at the societal level, whereas the operational, financial, and infection-prevention risks are carried internally by hospitals. This misalignment helps explain why initiatives often stall after pilots. Participants also rarely mentioned broader organizational co-benefits, such as supply chain resilience, improved employer reputation, and longer-term cost savings. Making these benefits explicit may help reframe circularity as a strategic opportunity rather than a moral add-on.

To our knowledge, this is the first study to examine how hospitals approach the circular transition. A key strength of our study is the use of an evidence-based framework to understand implementation in complex settings. An additional strength is the diversity of participants’ roles and participating hospitals in the Netherlands, a country where all hospitals signed a national healthcare sustainability agreement. Hence, although our sample is based in a specific region, we expect it to be largely representative of national developments. A limitation of this study is the absence of top-management perspectives. Although board members from three hospitals were invited, none participated. Moreover, the study’s generalizability may be limited by its focus on the Dutch context. The Netherlands has a decentralized healthcare system [40], and has a relatively advanced policy context for healthcare sustainability and circularity, including national commitments through the 'Green Deal 3.0', which includes broad stakeholder engagement [7]. While many of the identified barriers and facilitators are likely relevant across healthcare systems, the level of awareness, support, and momentum for circularity may differ in other national contexts.

## 5 Conclusion

Dutch hospitals are increasingly active in the circular transition, largely through bottom-up initiatives led by motivated staff and green teams. However, these efforts are typically fragmented and operate without consistent organizational support, budgets, or monitoring. Circularity differs fundamentally from conventional hospital change: its environmental benefits accumulate mainly at the societal level, while hospitals themselves bear the operational, financial, workflow-related, and infection-prevention risks. Addressing this misalignment is important for progress. Strengthening top-down engagement through leadership commitment, clear priorities, and dedicated resources are important to move beyond pilots. Embedding circularity into organizational processes, governance structures, policies, clinical procedures, and performance-monitoring systems can provide the stability and accountability needed for long-term implementation. System-level changes are needed as well, to address the absence of affordable circular alternatives, dependence on incumbent linear-economy suppliers, and to showcase co-benefits of circularity to not only hospital management, but also health insurers and regulatory bodies so that they can fine-tune their policies accordingly. Future research should explore how leadership, regulations, accreditation schemes, and performance measurement can best support hospitals in scaling circular practices, how markets for safe, affordable and genuinely sustainable products can be accelerated, and how supply chains and reusable circular ecosystems can be developed.

## Ethics approval and consent to participate

Ethical review was conducted by the non-WMO Committee of the Medical Ethics Review Committee of Amsterdam University Medical Center (IRB00013752, document number: 2025.0113), which concluded this study falls outside the scope of the Medical Research Involving Human Subjects Act (WMO; Wet Medisch-wetenschappelijk Onderzoek met mensen). Written or verbally-recorded informed consent was obtained prior to the interviews.

## Availability of data and materials

The datasets generated and/or analyzed during the current study are not publicly available due the sensitive nature of some of the answers to the questions asked in the interviews of this study.

## Funding

This study was funded through internal project funding of Amsterdam University Medical Centre.

## CRediT authorship contribution statement

**Maaike L. Soors d’Ancona:** Writing – review & editing, Writing – original draft, Visualization, Resources, Project administration, Methodology, Investigation, Formal analysis, Data curation, Conceptualization. **Óscar Brito Fernandes:** Writing – review & editing, Supervision, Resources, Project administration, Methodology, Conceptualization. **Ans Kolk:** Writing – review & editing, Supervision, Resources, Methodology, Conceptualization. **Lisanne H.J.A. Kouwenberg:** Writing – review & editing, Resources, Investigation. **Nicolaas H. Sperna Weiland:** Writing – review & editing, Supervision, Resources, Methodology, Funding acquisition, Conceptualization. **Dionne S. Kringos:** Writing – review & editing, Supervision, Resources, Methodology, Funding acquisition, Conceptualization.

## Declaration of competing interest

The authors declare that they have no known competing financial interests or personal relationships that could have appeared to influence the work reported in this paper.

## Footnotes

Supplementary material associated with this article can be found, in the online version, at doi:10.1016/j.joclim.2026.100727.
