# Supplementary material for: Towards circular healthcare: A qualitative exploration of approaches and implementation factors in Dutch hospitals
Source: J Clim Chang Health. 2026 Oct 1;32:100727. doi: 10.1016/j.joclim.2026.100727 (PMC13657179; doi:10.1016/j.joclim.2026.100727)
Supplement: Supplementary file 1 [file mmc1.docx]

# Appendix A. Interview guide (English and Dutch)

English (translated from Dutch)

**Introductory text**

*[introduction of interviewer]*

Thank you for participating in this interview for our research on circularity in hospitals.

Today I would like to hear your experiences about the implementation of circularity initiatives in your hospital. We want to use this information to create an overview of the approaches to circular healthcare of Dutch hospitals in the region of Amsterdam, and the strategies they use for the implementation of circularity initiatives. With circularity initiatives, I would like to focus on medical initiatives such as hospital waste reduction and reusable medical clothing, materials and equipment, and less on initiatives such as reusable drinking cups.

We also want to identify factors influencing the implementation of circularity initiatives. Factors we are looking for are all the things you experienced that facilitated or hindered the implementation process in your hospital. There are no right or wrong answers during this interview. Hospital nor participant names will be mentioned in the publication.

Do you consent to (this interview and) the recording of this conversation? This will allow us to better study the conversation at a later time so that no valuable information is lost. The content of this conversation will remain confidential. The recording will be stored digitally and only researchers will have access to it. Once the recording is no longer needed for the research, it will be deleted.

*[IF: written consent was obtained or the participant consents verbally]* ***Turn on recording***

Please repeat that you consent to the interview on the recording.

**Background information about the participant**

| *Topic* | *Questions* |
| --- | --- |
| Introduction participant | Could you please introduce yourself? Please state your name, role, and how long you have been working in your current role at this hospital.   - *Can you tell me more about your work within the hospital in relation to circularity?*   - [*If sustainability is a secondary function*]*: How long has sustainability been part of your work?* |

**Setting the scene for circularity**

| *Topic* | *Questions* |
| --- | --- |
| Circularity initiatives involved in | Can you tell me briefly about circularity initiatives that have been implemented in the hospital that you have been involved in?   - *[With little response, list example initiative(s) based on hospital’s website and/or documents]*   - *Is this correct?* |
| Non-implemented initiatives | Can you tell me briefly about circularity initiatives that you (or your colleagues) wished to but were not able to implement (yet)? |
| Current implementations | Can you tell me whether there are initiatives that are currently in the progress of being implemented?   - *If yes, could you elaborate on this/these?* |

**Process domain**

| *Topic* | *Questions* |
| --- | --- |
| Organization of transition | Can you describe how your hospital organizes the circular transition?   - *Do you have specific working groups or steering committees or does it depend more on the individual people (e.g. green teams) working on circularity?* - *What do you think of this approach?* |
| Implementation plan | Can you describe what the plan looked like when you started implementing a certain circularity initiative in your hospital? What activities, actions and strategies were used during the process?   - *How did you like this plan? Does this work well, in your opinion?* - *How does the strategy differ between different types of circularity initiatives?* |
| Facilitators | In your experience, what were factors that facilitated the implementation process of this initiative? |
| Barriers | In your experience, what were factors that hindered/slowed/impaired the implementation process?   - *Did you also encounter these hindering factors in other implementations? Or do they differ from one initiative to another?* |

**Individuals domain**

| *Topic* | *Questions* |
| --- | --- |
| Key individuals | Who were the key individuals in your hospital to get on board with implementation of circularity initiatives? Please elaborate.   - *What are their roles?* |
| Traits of individuals | What makes these individuals important for implementation?  What characteristics do they possess? |

**Innovation domain**

| *Topic* | *Questions* |
| --- | --- |
| Decision-making process | What does your decision-making process for circularity initiatives look like? |
| Characterization of chosen initiatives | Why has your hospital chosen the specific circularity initiatives that it has implemented? What makes these initiatives the “chosen ones”? |

**Inner setting domain**

| *Topic* | *Questions* |
| --- | --- |
| Internal collaboration | With whom within the hospital do you collaborate the most when it comes to circularity?   - *With whom within the hospital would you like to collaborate (more)?*   - *Why?* |
| Culture | How has the culture of your hospital (the values, norms, and beliefs shared throughout the organization) influenced the implementation of circularity initiatives? |
| Receptivity to initiatives | How did you perceive the general level of receptivity in your hospital to implement [certain initiative]?   - *To what extent is this the case for other circularity initiatives in your hospital?* |
| *Constructs specific to the implementation* | |
| Compatibility | How did [certain initiative] integrate into existing work processes? To what extent does circularity fit into existing work processes?   - *Were changes of the initiative necessary in order to fit with existing work processes? If so, what changes?* - *Were changes of work processes in the hospital needed in order to fit with the initiative? If so, what changes?* |
| Incentive systems | Were there any tangible and/or intangible incentives or rewards within the hospital that supported implementations? *(E.g., tangible; cost savings through reuse, financial incentives/subsidies, more efficient work processes, intangible: reputation, recognition and appreciation, positive work culture, patient perception, societal impact)* |
| Mission alignment | To what extent does implementing circularity align with your hospital’s mission and goals? |
| Available resources | To what extent were the necessary resources (e.g. funding, space, materials & equipment) available to implement the initiative?   - *To what extent is this the case for other circularity initiatives in your hospital?* |

**Outer setting domain**

| *Topic* | *Questions* |
| --- | --- |
| External collaboration | With whom outside the hospital do you collaborate the most when it comes to circularity?   - *With whom outside the hospital would you like to collaborate (more)? Why?* |
| Collaboration with other hospitals | Can you describe how connections and collaboration with other hospitals affected the implementation of [certain initiative]? Were there any specific partnerships, knowledge exchanges or joint projects that played a role in this?   - *What about other circularity initiatives in your hospital?* |
| Collaboration with supply-chain | In what way did supply-chain influence the implementation of [certain initiative]? Can you give examples of how communication and cooperation with these parties contribute to or actually hinder the circular transition?   - *What about other circularity initiatives in your hospital?* |
| External pressure | What kinds of pressure from outside the hospital do you think influences the implementation of circularity? (E.g. social pressure, politics, media campaigns, competition from other hospitals, performance indicators)   - *Which pressures positively influence implementation?* - *Which pressures negatively influence implementation?* - *What external pressures are missing, or would you like to see more of?* |

**Closing**

| *Topic* | *Questions* |
| --- | --- |
| Closing question | Looking ahead, do you believe the hospital will continue on its current path toward circularity, or do you think other changes or strategies (or something else) will be necessary to scale up?  What do you see as the key steps or challenges in making further progress towards a fully circular hospital? |
| Add or clarify | Is there anything more you would like to add or clarify? |
| Snow-ball sampling | Who else in your hospital do you recommend that we talk to as well? |

Thank you for participating in this interview!

***Stop recording***

After transcribing this interview, I will email you the key insights and possibly some quotes. You can then confirm whether you find this a good reflection of the conversation, and if not, you can provide me with any corrections or additions.

Dutch

**Introductie tekst**

*[introductie van interviewer]*

Bedankt dat je mee wilt doen aan dit interview voor ons onderzoek over circulariteit in ziekenhuizen.

Vandaag wil ik graag je ervaringen horen over de implementatie van circulariteitsinitiatieven in jouw ziekenhuis. Aan de hand van deze informatie willen we een overzicht maken van hoe Nederlandse ziekenhuizen in de regio Amsterdam de circulaire transitie organiseren, en de strategieën die ze gebruiken voor de implementatie van circulariteits-initiatieven. Met circulariteits-initiatieven wil ik graag focussen op medische initiatieven zoals ziekenhuisafval verminderen en herbruikbare medische kleding, materialen en apparatuur, en minder op initiatieven zoals herbruikbare drinkbekers.

Ook willen we factoren identificeren die van invloed zijn op de implementatie van circulariteits-initiatieven. Factoren waar we naar op zoek zijn, zijn alle dingen die je hebt ervaren die het implementatieproces in jullie ziekenhuis vergemakkelijkt of belemmert. Er zijn geen goede of foute antwoorden tijdens dit interview. Namen van ziekenhuizen en van de mensen die we interviewen worden niet genoemd in de uiteindelijke publicatie.

Geef je toestemming voor (het interview en) het opnemen van dit gesprek? Dan kunnen we het gesprek op een later moment beter bestuderen zodat er geen waardevolle informatie verloren gaat. De inhoud van dit gesprek blijft vertrouwelijk. De opname wordt beschermd digitaal opgeslagen en alleen onderzoekers hebben er toegang toe. Wanneer de opname niet langer nodig is voor het onderzoek wordt deze verwijderd.

*[INDIEN: schriftelijke toestemming is verkregen of de deelnemer mondelinge toestemming geeft]* ***Zet opname aan***

Herhaal alsjeblieft dat je toestemming geeft voor het interview op de opname.

**Achtergrondinformatie van deelnemer**

| *Onderwerp* | *Vragen* |
| --- | --- |
| Introductie deelnemer | Wil je je voorstellen? Vermeld alsjeblieft je naam, functie, en hoe lang je al in jouw huidige functie in dit ziekenhuis werkt.   - *Kan je mij meer vertellen over je werkzaamheden binnen het ziekenhuis met betrekking tot circulariteit?*   - [*Indien duurzaamheid een nevenfunctie is*]*: Hoe lang maakt duurzaamheid deel uit van je werkzaamheden?* |

**De toon zetten voor circulariteit**

| *Onderwerp* | *Vragen* |
| --- | --- |
| Circulariteits-initiatieven bij betrokken geweest | Kan je mij kort vertellen over een aantal circulariteits-initiatieven die in je ziekenhuis zijn geïmplementeerd waar je bij betrokken bent geweest?   - *[Bij weinig respons, noem voorbeeldinitiatieven op basis van website en/of documenten van het ziekenhuis]*   - *Klopt dit?* |
| Niet geïmplementeerde initiatieven | Kan je mij kort vertellen over circulariteits-initiatieven die jij (of je collega’s) had willen, maar (nog) niet hebt kunnen implementeren? |
| Huidige implementaties | Zijn er initiatieven die op dit moment worden geïmplementeerd?   - *Zo ja, kan je hier wat meer over vertellen?* |

**Proces domein**

| *Onderwerp* | *Vragen* |
| --- | --- |
| Organisatie van transitie | Kan je beschrijven hoe jullie ziekenhuis de circulaire transitie organiseert?   - *Hebben jullie bepaalde werkgroepen of stuurgroepen of hangt het meer op de losse personen (bijv. green teams) die zich inzetten voor circulariteit?* - *Wat vind je van deze aanpak?* |
| Implementatieplan | Kan je omschrijven hoe het plan eruit zag wanneer jullie een bepaald circulariteits-initiatief gingen implementeren in jouw ziekenhuis? Welke acties en strategieën werden gebruikt tijdens het proces?   - *Hoe beviel dit plan? Werkt dit goed, naar jouw idee?* - *Hoe verschilt de strategie tussen verschillende soorten circulariteits-initiatieven?* |
| Bevorderende factoren | Wat waren in jouw beleving factoren die het implementatieproces van dit initiatief bevorderde/vergemakkelijkte? |
| Belemmerende factoren | Wat waren in jouw beleving factoren die het implementatieproces belemmerde / vertraagde / vermoeilijkte?   - *Trof je deze belemmerende factoren ook bij andere implementaties? Of verschilt het per initiatief?* |

**Individu domein**

| *Onderwerp* | *Vragen* |
| --- | --- |
| Belangrijke personen | Wie waren de belangrijke personen in jouw ziekenhuis die aan boord moesten komen met de implementatie van circulariteits-initiatieven? Graag nader toelichten.   - *Wat zijn hun rollen/functies?* |
| Eigenschappen van personen | Wat maakt deze personen belangrijk voor het implementeren?  Welke eigenschappen bezitten ze? |

**Innovatie domein**

| *Onderwerp* | *Vragen* |
| --- | --- |
| Besluitvormingsproces | Hoe ziet jullie besluitvormingsproces voor circulariteits-initiatieven eruit? |
| Eigenschappen van gekozen initiatieven | Waarom heeft jouw ziekenhuis gekozen voor de specifieke circulariteits-initiatieven die zijn geïmplementeerd? Wat maakt deze initiatieven de “uitverkorenen”? |

**Inner setting domein**

| *Onderwerp* | *Vragen* |
| --- | --- |
| Interne samenwerkingen | Met wie binnen het ziekenhuis werk je het meeste samen over circulariteit?   - *Met wie binnen het ziekenhuis zou je graag (meer) willen samenwerken?*   - *Waarom?* |
| Cultuur | Hoe heeft de cultuur van jullie ziekenhuis (waarden, normen en overtuigingen binnen de hele organisatie) de implementatie van circulariteits-initiatieven beïnvloed? |
| Bereidheid voor initiatieven | Hoe ervaarde je de algemene bereidheid in jullie ziekenhuis om [bepaald initiatief] te implementeren?   - *In hoeverre is dat het geval voor andere circulariteits-initiatieven in jullie ziekenhuis?* |
| *Constructen specifiek voor de implementatie* | |
| Compatibiliteit | Hoe integreerde [bepaald initiatief] in de bestaande werkprocessen? In hoeverre past circulariteit in bestaande werkprocessen?   - *Moest er iets veranderd worden aan het initiatief om aan te sluiten bij bestaande werkprocessen? Zo ja, wat dan?* - *Moesten er werkprocessen in het ziekenhuis veranderen om aan te sluiten bij het initiatief? Zo ja, wat dan?* |
| Stimulansen / beloningen | Waren er materiële en/of immateriële stimulansen of beloningen binnen het ziekenhuis die de implementaties ondersteunden? *(Bijv., materieel; kostenbesparing door hergebruik, financiële prikkels/subsidies, efficiëntere werkprocessen, immaterieel: reputatie, erkenning en waardering, positieve werkcultuur, patiënten-perceptie, maatschappelijke impact)* |
| Missie aansluiting | In welke mate sluit de implementatie van circulariteit aan bij de missie en doelen van jullie ziekenhuis? |
| Beschikbaarheid benodigde middelen | In welke mate waren de benodigde middelen (bijv. financiering, ruimte, materialen & apparatuur) beschikbaar om het initiatief te implementeren?   - *In hoeverre is dat het geval voor andere circulariteits-initiatieven in jullie ziekenhuis?* |

**Outer setting domein**

| *Onderwerp* | *Vragen* |
| --- | --- |
| Externe samenwerkingen | Met wie buiten het ziekenhuis werk je het meeste samen als het gaat om circulariteit?   - *Met wie buiten het ziekenhuis zou je graag (meer) willen samenwerken? Waarom?* |
| Samenwerkingen met andere ziekenhuizen | Kan je beschrijven hoe de connecties en samenwerking met andere ziekenhuizen een invloed had op jullie implementatie van [bepaald initiatief]? Waren er specifieke samenwerkingsverbanden, kennisuitwisselingen of gezamenlijke projecten die hierin een rol speelden?   - *Hoe zit dat met andere circulariteitsinitiatieven in jullie ziekenhuis?* |
| Samenwerkingen met ketenpartners | Op welke manier beïnvloedden leveranciers en ketenpartners de implementatie van [bepaald initiatief]? Kan je voorbeelden geven van hoe communicatie en samenwerking met deze partijen bijdragen aan of juist belemmeringen vormen voor de circulaire transitie?   - *Hoe zit dat met andere circulariteits-initiatieven in jullie ziekenhuis?* |
| Externe druk | Wat voor soort druk van buiten het ziekenhuis heeft volgens jou invloed op jullie implementatie van circulariteit? (Bijv. maatschappelijke druk, politiek, campagnes in de media, concurrentie van andere ziekenhuizen, prestatie-indicatoren)   - *Welke druk beïnvloedt implementatie positief?* - *Welke druk beïnvloedt de implementatie negatief?* - *Welke druk van buitenaf ontbreekt er, of zou je meer van willen zien?* |

**Afsluiting**

| *Onderwerp* | *Vragen* |
| --- | --- |
| Afsluitende vraag | Denk je dat jullie ziekenhuis op de huidige weg doorgaat richting circulariteit, of zijn er volgens jou andere veranderingen of strategieën (of iets anders) nodig om verder op te schalen?  Wat zie je als de belangrijkste stappen of uitdagingen om verdere vooruitgang te boeken richting een volledig circulair ziekenhuis? |
| Toevoegen of verhelderen | Is er nog iets dat je wilt toevoegen of verhelderen? |
| Snow-ball sampling | Met wie binnen jullie ziekenhuis zouden we nog meer moeten praten? |

Hartelijk dank voor deelname aan dit interview!

***Zet opname uit***

Na het transcriberen van dit interview zal ik de belangrijkste inzichten en eventueel enkele quotes mailen. Dan kan je bevestigen of dit een goede weerspiegeling van het gesprek vindt, en zo niet, kan je mij verbeteringen of aanvullingen geven.

# Appendix B. Consolidated Framework for Implementation Research (CFIR) domains

The framework’s five domains − intervention characteristic, inner setting, outer setting, characteristics of individuals, and process of implementation – represented the basis for question development. The intervention characteristic domain refers to perceptions of the circular initiatives. The outer setting domain concerns perceptions of the setting in which the hospital operates, including regulatory bodies, professional networks, societal expectations and the supply-chain. The inner setting domain covers perceptions of the hospital and its departments, where circular initiatives are implemented. The characteristics of individuals domain is defined as the characteristics and perceptions of individuals involved in the circular initiatives. The implementation process domain entails the activities and strategies used to approach the circular transition and to implement and deliver circular initiatives.

# Appendix C. Consolidated criteria for reporting qualitative studies (COREQ)

Table C.1 Consolidated criteria for reporting qualitative studies (COREQ): 32-item checklist

| **No** | **Item** | **Guide questions/description** | **Remarks** | **Remarks** |
| --- | --- | --- | --- | --- |
| **Domain 1: Research team and reflexivity** | | | | |
| Personal Characteristics | | | | |
| 1. | Interviewer/facilitator | Which author/s conducted the interview or focus group? | One author (MS) conducted the interviews. One of the researchers (LK) joined the first 3 interviews in order to provide feedback afterwards. |  |
| 2. | Credentials | What were the researcher's credentials? *E.g. PhD, MD* | MS: MSc  OBF: PhD  AK: Prof, PhD  LK: MD, MSc  NSW: MD, PhD  DK: Prof, PhD |  |
| 3. | Occupation | What was their occupation at the time of the study? | MS: PhD Candidate  OBF: Assistant professor  AK: Full professor  LK: PhD Candidate  NSW: Academic, anesthesiologist  DK: Full professor |  |
| 4. | Gender | Was the researcher male or female? | MS: female  OBF: male  AK: female  LK: female  NSW: male  DK: female |  |
| 5. | Experience and training | What experience or training did the researcher have? | LK attended a course on “Qualitative Health Research”.  OBF, AK, NSW, and DK are experienced researchers in qualitative studies and have collectively published several qualitative research articles. |  |
| Relationship with participants | | | | |
| 6. | Relationship established | Was a relationship established prior to study commencement? | No. The researchers knew one of the participants as this participant is part of the research team. As this person was involved in the preparation of this study, the participation in one of the interviews may have been influenced by previous discussions. |  |
| 7. | Participant knowledge of the interviewer | What did the participants know about the researcher? e*.g. personal goals, reasons for doing the research* | Two participants knew the interviewer (MS) professionally. At least one of the remaining participants knew NSW professionally, who did not conduct interviews. All participants knew the reason for doing the research, as this was stated in the Informed Consent form and explained prior to the interviews. |  |
| 8. | Interviewer characteristics | What characteristics were reported about the interviewer/facilitator? e.g. *Bias, assumptions, reasons and interests in the research topic* | None. |  |
| **Domain 2: study design** | | | | |
| Theoretical framework | | | | |
| 9. | Methodological orientation and Theory | What methodological orientation was stated to underpin the study? *e.g. grounded theory, discourse analysis, ethnography, phenomenology, content analysis* | Thematic analysis. |  |
| Participant selection | | | | |
| 10. | Sampling | How were participants selected? *e.g. purposive, convenience, consecutive, snowball* | Purposive sampling and snowball sampling were used to select participants. An information sheet explaining the purpose of the study was sent to potential participants. | See 2.3 Participant selection |
| 11. | Method of approach | How were participants approached? e*.g. face-to-face, telephone, mail, email* | Email. | See 2.3 Participant selection |
| 12. | Sample size | How many participants were in the study? | 19 participants. | See Table 1. |
| 13. | Non-participation | How many people refused to participate or dropped out? Reasons? | 1 person refused to participate but referred us to a close colleague to interview. 1 person initially dropped out prior to the planned interview due to availability, but was open to answer a subset of role-specific interview questions via email and was therefore included in the study, categorized into online participation. The members of the board of directors of 3 different hospitals were invited to participate but ignored the invitation. |  |
| Setting | | | | |
| 14. | Setting of data collection | Where was the data collected? e*.g. home, clinic, workplace* | Data were collected through interviews via Microsoft Teams or at the hospital where the participant worked. |  |
| 15. | Presence of non-participants | Was anyone else present besides the participants and researchers? | One interview was held in a corner of a quiet hospital cafeteria. Otherwise no. |  |
| 16. | Description of sample | What are the important characteristics of the sample? *e.g. demographic data, date* | Role and hospital of employment. |  |
| Data collection | | | | |
| 17. | Interview guide | Were questions, prompts, guides provided by the authors? Was it pilot tested? | An interview guide was prepared and pilot tested with one participant, being part of the research team. Data collected in the interview were included in the final analysis. | See Appendix A |
| 18. | Repeat interviews | Were repeat interviews carried out? If yes, how many? | No. |  |
| 19. | Audio/visual recording | Did the research use audio or visual recording to collect the data? | Interviews were audio recorded, sometimes with video. |  |
| 20. | Field notes | Were field notes made during and/or after the interview or focus group? | Yes, to inform subsequent interview questions and to keep track of the interview guide. |  |
| 21. | Duration | What was the duration of the interviews or focus group? | The duration of the interviews ranged from 29 to 68 minutes. |  |
| 22. | Data saturation | Was data saturation discussed? | Yes, in the methods section. | See 2.1 Study design and data collection |
| 23. | Transcripts returned | Were transcripts returned to participants for comment and/or correction? | No. Key insights and potential quotes were shared with participants after transcription via email for comment and/or correction. The majority of interview insights were confirmed by participants to accurately reflect the interviews. Adjustments to the insights mainly included the addition of details on the discussed topics, including specificities of initiatives, knowledge-sharing agendas, and decision-making processes. |  |
| **Domain 3: analysis and findings** | | | | |
| Data analysis | | | | |
| 24. | Number of data coders | How many data coders coded the data? | Two researchers (MS, LK) independently coded the first two interviews, whilst one researcher (MS) coded the remaining interviews. | See 2.4 Interview conduct |
| 25. | Description of the coding tree | Did authors provide a description of the coding tree? | No. |  |
| 26. | Derivation of themes | Were themes identified in advance or derived from the data? | Themes were derived from the data. |  |
| 27. | Software | What software, if applicable, was used to manage the data? | MAXQDA 2024 [1] and Microsoft Excel. |  |
| 28. | Participant checking | Did participants provide feedback on the findings? | Key insights and potential quotations were sent to participants via email for corrections and validation. | See 2.4 Interview conduct |
| Reporting | | | | |
| 29. | Quotations presented | Were participant quotations presented to illustrate the themes / findings? Was each quotation identified? e*.g. participant number* | Yes, quotations were presented and identified by the participant number and role. | See 2.4 Interview conduct |
| 30. | Data and findings consistent | Was there consistency between the data presented and the findings? | Yes. |  |
| 31. | Clarity of major themes | Were major themes clearly presented in the findings? | Yes. |  |
| 32. | Clarity of minor themes | Is there a description of diverse cases or discussion of minor themes? | Yes. |  |

# Appendix D. Complete table of barriers and facilitators

Table D.1 Implementation barriers and facilitators identified by the interviewed hospital employees, categorized using the updated CFIR and organized into 8 overarching themes

| **CFIR categories of implementation factors** | | **Barriers** | | **Facilitators** | |  |
| --- | --- | --- | --- | --- | --- | --- |
| **THEME 1: Resource availability** | | | | | |  |
| *Innovation domain* | | | | | |  |
| Innovation evidence-base | |  | | Making a strong business case of the initiative. | |  |
| Innovation complexity | | Shared-savings system’s issues. | |  | |  |
| Innovation cost | | Initiatives often being expensive to implement. | |  | |  |
| *Outer setting domain* | |  | |  | |  |
| Critical incidents | |  | | Increased awareness of material scarcity due to COVID-19 pandemic. | |  |
| Local conditions | | Shortage of healthcare workers. | |  | |  |
|  | | Education of nurses focused on frequent disposable use. | |  | |  |
| Partnerships & connections | | LinkedIn hospital posts about initiatives not being reliable. | | Communication with other hospitals to share info. | |  |
|  | |  | | Gathering inspiration and advice from online platforms. | |  |
|  | |  | | Communication with national committees to share information. | |  |
| Financing | | Applying for grants being difficult and time-intensive. | | Obtaining grants for circularity. | |  |
| External pressure | |  | |  | |  |
| *Market pressure* | |  | | Sustainability as potential advantage for attracting personnel. | |  |
| *Inner setting domain* | |  | |  | |  |
| Structural characteristics | |  | |  | |  |
| *Physical infrastructure* | | Shortage of physical space for reusables or waste separation. | | More space created for waste separation during construction. | |  |
| *Work infrastructure* | | Receiving too little protected hours for sustainability. | | Presence of a sustainability coordinator. | |  |
|  | | Capacity problems among support services. | | Receiving dedicated hours for sustainability. | |  |
|  | | Financial problems. | | Having an assortment coordinator to optimize space use. | |  |
|  | | Absence of a sustainability coordinator. | |  | |  |
| Communications | |  | | Organized meet-ups with green teams for knowledge-sharing. | |  |
|  | |  | | Increasing circularity visibility through communication in the hospital. | |  |
|  | |  | | Use of digital platforms (e.g. Teams) between green teams. | |  |
| Incentive systems | | No (im)material incentives or rewards for efforts. | | Acknowledging and/or rewarding efforts. | |  |
| Available resources | | Not receiving (structural) sustainability budget. | | Receiving a sustainability budget. | |  |
|  | | Departments unwilling to transfer saved money to sustainability budget. | |  | |  |
| Access to knowledge & information | | Assumptions that sustainability is always more expensive. | | Creating understanding and awareness about circularity. | |  |
|  | | Missing vision on definition of a circular hospital. | |  | |  |
|  | | Having limited expertise on circularity in the workplace. | |  | |  |
|  | | Difficult to do awareness/exposure in the hospital. | |  | |  |
|  | | Reaching the hard-to-reach is difficult. | |  | |  |
| *Individuals domain* | |  | |  | |  |
| Mid-level leaders (department heads) | |  | | Manager able to secure sustainability budget. | |  |
| Implementation team members (green team, coordinator, etc.) | | Not enough resources to implement big projects bottom-up. | |  | |  |
| Innovation recipients (healthcare workers) | | High workload of healthcare staff. | |  | |  |
| *Implementation process domain* | | | |  | |  |
| Adapting | |  | | Procuring a waste compactor to facilitate waste infrastructure. | |  |
| **THEME 2: Implementation process** | | | | |  | |
| *Outer setting domain* |  | |  | |  | |
| Critical incidents |  | | The COVID-19 pandemic circumstances fastened implementation procedures. | |  | |
| *Implementation process domain* | | | | |  | |
| Assessing needs |  | | Timely involvement of relevant internal stakeholders in the implementation process. | |  | |
| Planning |  | | Using a structured implementation plan. | |  | |
| Tailoring strategies |  | | Choosing to still provide old (disposable) product for back-up. | |  | |
|  |  | | Having a clear decision-making and implementation structure. | |  | |
| Engaging |  | | Ensuring repeated exposure to the (upcoming) initiative. | |  | |
|  |  | | Not forcing initiatives on employees. | |  | |
| *Innovation recipients* | Resistance towards implementation after non-engagement during implementation process. | |  | |  | |
| Doing | Slow implementation process impairing initial enthusiasm. | | Piloting the initiative in the user group. | |  | |
|  | Doing too many things at once causing delays in the implementation processes. | | Choosing to implement radically; costing less energy and creating more impact more quickly. | |  | |
|  | Desired high speed of green team not matching infection prevention specialist workload. | | Fast implementation giving energy for green team members. | |  | |
|  |  | | Using a workflow for green teams to help embed the initiative in the organization. | |  | |
| **THEME 3: Circular initiative characteristics** | | | | |  | |
| *Innovation domain* |  | |  | |  | |
| Innovation source |  | | Idea originating from green teams. | |  | |
| Innovation evidence-base | Questionable sustainability impact, either due to missing, questionable or complex data. | | Data proving the sustainability impact. | |  | |
| Innovation relative advantage |  | | Initiative matching or simplifying the existing workflows. | |  | |
|  |  | | Initiative matching personal preferences. | |  | |
|  |  | | Highly visible initiatives. | |  | |
| Innovation adaptability |  | | Possibility for partial implementation. | |  | |
|  |  | | Possibility to adapt the initiative to user needs. | |  | |
| Innovation complexity | Certain circular initiatives requiring more time and effort to implement. | |  | |  | |
| Innovation design | Certain initiatives being less user friendly. | |  | |  | |
| Innovation cost |  | | Initiative saving costs. | |  | |
|  |  | | Initiative same price. | |  | |
| *Inner setting domain* |  | |  | |  | |
| Culture | Affecting “personal expression/identity” | |  | |  | |
| Compatibility | Initiative involving unfavorable changes in workflows. | |  | |  | |
| Relative priority | Volume needed for business case. | |  | |  | |
| **THEME 4: Staff and leadership motivation, engagement and attitudes** | | | | | |  |
| *Innovation domain* | |  | |  | |  |
| Innovation relative advantage | |  | | Older employees remembering reusable medical instruments. | |  |
|  | |  | | Framing circularity as preventing wastage. | |  |
| *Outer setting domain* | |  | |  | |  |
| Local attitudes | |  | | Momentum around sustainability both within and outside healthcare. | |  |
| Partnerships & connections | |  | | Meetings with “green” colleagues for motivation. | |  |
| External pressure | |  | |  | |  |
| *Societal pressure* | |  | | Wanting to publicly show progress. | |  |
| *Market pressure* | |  | | Being the first hospital with an initiative. | |  |
|  | |  | | Wanting to keep up with other hospitals. | |  |
| *Inner setting domain* | |  | |  | |  |
| Structural characteristics | |  | |  | |  |
| *Work infrastructure* | | Insufficient top-down control, guidance or support for sustainability. | |  | |  |
| Relational connections | | Interdepartmental distrust towards sterilization. | | Good cohesion of sustainability department with other departments. | |  |
| Culture | | Sustainability not part of organizations (corporate) culture. | |  | |  |
| Relative priority | | Sustainability not prioritized, or having too many other priorities and/or changes. | |  | |  |
| *Individuals domain* | |  | |  | |  |
| High-level leaders (board of directors) | | Non-proactive behavior or not enough engagement of board on sustainability. | | Support from board of directors. | |  |
| Mid-level leaders (department heads) | | Lack of motivation/support towards sustainability changes from department heads. | | Motivated/supportive department heads towards sustainability. | |  |
|  | | Management must agree for implementation. | |  | |  |
| Implementation facilitators (support services) | | Lack of motivation towards sustainability changes from support services. | | Supportive for sustainability changes from support services. | |  |
|  | |  | | Having good connections with facility staff. | |  |
| Implementation leads (leading figures) | |  | | Having an individual champion in the workplace. | |  |
|  | |  | | Continuing efforts despite resistance in the workplace. | |  |
| Implementation team members (green team, coordinator, etc.) | | Losing interest or perseverance due to long implementation process. | | Motivation towards sustainability changes. | |  |
|  | | Demotivation due to dismissive/negative remarks from colleagues. | | Having patience. | |  |
|  | |  | | Being motivated by challenge. | |  |
|  | |  | | Feeling supported by colleagues. | |  |
|  | |  | | Energy investments of sustainability coordinator paying off. | |  |
|  | |  | | Many people working on initiatives. | |  |
| Innovation recipients (healthcare workers) | | Resistance to change in healthcare staff. | | Motivation and support for sustainability changes. | |  |
|  | | Sustainability being seen as higher workload. | | Younger generation being more interested in sustainability. | |  |
|  | | Skepticism of impact of initiatives. | |  | |  |
|  | | Older generation being less interested in sustainability. | |  | |  |
|  | | A long time to communicate achievements decreasing enthusiasm. | |  | |  |
|  | | Nurses feeling incapable for implementation of changed procedures. | |  | |  |
| *Implementation process domain* | | | | | |  |
| Tailoring strategies | |  | | Frequent communicating circular progress to increase support. | |  |
| Engaging | |  | | Creating competition to engage healthcare staff. | |  |
|  | |  | | Sustainability coordinator contributing ideas for solutions on how to change workflows. | |  |
|  | |  | | Approaching and/or framing initiatives positively. | |  |
| **THEME 5: Tension between infection prevention and circularity** | | | | | |  |
| *Innovation domain* | |  | |  | |  |
| Innovation evidence-base | |  | | Data proving the safety of more sustainable products. | |  |
| Innovation complexity | | Initiatives in a high-risk context being complex from an infection prevention perspective. | | Refuse/reduce being easier to implement than reuse initiatives. | |  |
| Innovation design | | Innovation perceived as not safe enough by infection prevention specialists. | |  | |  |
| *Outer setting domain* | |  | |  | |  |
| Policies & laws | | Missing ISO norms for some reusable products, inhibiting the persuasion of infection prevention specialists. | | Updated guideline facilitating circularity. | |  |
|  | | Lack of/unfavorable guidelines on circular workflows. | |  | |  |
|  | | Guidelines not being binding. | |  | |  |
| *Inner setting domain* | |  | |  | |  |
| Structural characteristics | |  | |  | |  |
| *Work infrastructure* | | Infection prevention protocol and guideline restrictions. | |  | |  |
|  | | Regional hospital not generating research, thus depending more on national guidelines. | |  | |  |
| Access to knowledge & information | | Staff having insufficient knowledge on infection prevention safety of initiatives. | |  | |  |
| *Individuals domain* | |  | |  | |  |
| Implementation facilitators (support services) | | Different approach to risks between doctors and infection prevention specialists. | | “Green” infection prevention specialists. | |  |
|  | |  | | Preference of infection prevention specialist matching the user preference. | |  |
| *Implementation process domain* | | | | | |  |
| Assessing needs | | Involving infection prevention specialists late in the implementation process. | |  | |  |
| **THEME 6: Organizational complexity** | | | | | |  |
| *Innovation domain* | |  | |  | |  |
| Innovation complexity | | Projects often involving many stakeholders. | |  | |  |
| *Inner setting domain* | |  | |  | |  |
| Structural characteristics | |  | |  | |  |
| *Physical infrastructure* | | Large hospital, making implementation more difficult. | | Small hospital with shorter lines of communication. | |  |
|  | | Renovation of infrastructure. | |  | |  |
| *Work infrastructure* | | Hospitals being complex organizations. | | Having your own sterilization facility, thus less dependency. | |  |
|  | | Having little decision making power. | | Sustainability becoming integral part of regular work. | |  |
|  | | Frequent changes of personnel. | |  | |  |
|  | | Wanting to implement initiatives only hospital-wide, meaning everyone has to agree to the change. | |  | |  |
|  | | Imposing not fitting the hospital environment. | |  | |  |
|  | | Merger of departments leaving little time for sustainability. | |  | |  |
|  | | Structure of facilities department hindering attention to sustainability. | |  | |  |
| Compatibility | | Physical infrastructure not suitable for circular changes in workflows. | |  | |  |
| *Individuals domain* | |  | |  | |  |
| Implementation facilitators (support services) | | Sterilization unreliability hindering transition to reusable equipment. | |  | |  |
| **THEME 7: Regulations, certification and policy** | | | | |  | |
| *Outer setting domain* |  | |  | |  | |
| Local conditions | Political direction not in favor of sustainability. | |  | |  | |
| Policies & laws | Laws and regulations hindering circular transition. | | (Future) regulatory/policy changes triggering sustainable changes. | |  | |
|  | Dependency on professional groups for initiative approval. | | ‘Green deal 3.0’ stimulating transition. | |  | |
|  | Medical clothing obtained via lease contracts and therefore having to meet several requirements. | | Governmental waste policy facilitating hospitals’ waste separation. | |  | |
| External pressure |  | |  | |  | |
| *Performance-measurement pressure* |  | | Sustainability added to hospital performance indicators. | |  | |
|  |  | | Certification incentivizing sustainability efforts. | |  | |
| *Inner setting domain* |  | |  | |  | |
| Structural characteristics |  | |  | |  | |
| *Work infrastructure* | Sustainability policy or strategy not acted upon enough. | |  | |  | |
| Mission alignment |  | | Having a clear sustainability purpose or goal. | |  | |
| **THEME 8: External stakeholders and suppliers** | | | | |  | |
| *Outer setting domain* |  | |  | |  | |
| Local conditions | Scale necessary for affordability. | | Suppliers willing to address sustainability. | |  | |
|  | Suppliers failing or unwilling to provide clear info on environmental benefit of products. | | Higher volume making sustainability interesting for suppliers. | |  | |
|  | Greenwashing. | |  | |  | |
|  | Disposable suppliers often not being reusable suppliers. | |  | |  | |
|  | Complex for suppliers to change, e.g. from disposable to reusable. | |  | |  | |
|  | Suppliers non pro-active attitude towards sustainability. | |  | |  | |
|  | Limited options in textile leading to high prices due to market dominance. | |  | |  | |
|  | Disposable suppliers decreasing prices. | |  | |  | |
|  | Waste processing company not accepting certain waste streams. | |  | |  | |
|  | External laundry capacity issues. | |  | |  | |
|  | Hospitals having many existing contracts in procurement. | |  | |  | |
|  | Circularity being dependent on medical developments. | |  | |  | |
|  | Medical advancements often being less environmentally friendly. | |  | |  | |
|  | Distrust in external sterilization department about reusables. | |  | |  | |
| Partnerships & connections | Dependency on supply-chain for transition, especially when switching from disposables to reusables. | | Leveraging hospital connections for more market power. | |  | |
|  | Not engaging in conversations with suppliers. | | Getting in contact with supply-chain for sustainable solutions. | |  | |
| Policies & laws | Municipality waste policy changes. | | Development of certification of suppliers to prevent greenwashing. | |  | |
|  | Market force difficulties due to different supplier contracts between hospitals. | |  | |  | |
|  | Not having enough scale for waste processing company to accept extra waste streams. | |  | |  | |
| *Implementation process domain* | | |  | |  | |
| Tailoring strategies |  | | Highlighting long-term economic benefit of the transition to reusable for suppliers. | |  | |

Abbreviations: CFIR, Consolidated Framework for Implementation Research; ISO, International Organization for Standardization.

**References**

1.	VERBI Software. MAXQDA 2024 [computer software]. Berlin, Germany: VERBI Software; 2024.
